# Changes and Appointments in Medical Schools

**Published:** 1858-09

**Authors:** 


### Editors' Table.

Changes and Appointments in Medical Schools.—Dr. J. H. B. McClellan, of this city, has been appointed to the Chair of Anatomy in the Pennsylvania Medical College, rendered vacant by the resignation of Professor Richardson. Dr. McClellan is the oldest son of the late Dr. George McClellan, the eminent surgeon, and principal founder of the Pennsylvania College. We congratulate the School upon the accession to its faculty of a gentleman of such distinguished *prestige* and acknowledged ability.

Dr. Eugene F. Colzey has been appointed to the Chair of Physiology in the Oglethorpe Medical College, at Savannah, Georgia.

Dr. Benson, of Chicago, has been elected Professor of Anatomy in the University of Louisville. This Chair had been rendered vacant by the transfer of Prof. Palmer to the Chair of Surgery. Dr. Lewis Rogers has resigned the Professorship of Clinical Medicine in the same institution, and Dr. Bemiss, our valued collaborator, has been appointed as his successor.

